# Study of a recombinant CHO cell line producing a monoclonal antibody by ATF or TFF external filter perfusion in a WAVE Bioreactor™

**DOI:** 10.1186/1753-6561-5-S8-P105

**Published:** 2011-11-22

**Authors:** Marie-Françoise Clincke, Carin Mölleryd, Ye Zhang, Eva Lindskog, Kieron Walsh, Véronique Chotteau

**Affiliations:** 1 School of Biotechnology, Animal Cell Technology Group, Royal Institute of Technology (KTH), SE-106 91 Stockholm, Sweden; 2 GE Healthcare Bio-Sciences AB, Björkgatan 30, SE-75184 Uppsala, Sweden; 3 GE Healthcare Bio-Sciences Corp, 14 Walk Up Drive, Westborough MA, 01581, USA

## Background

Major advantages of perfusion are high cell numbers and high total production in a relatively small size bioreactor. Moreover, perfusion is optimal when the product of interest is unstable or if the product yield is low. On the other hand, disadvantages are for example technical challenges originating from non-robust cell separation devices as well as sterility concerns from the more complex set-up needed.

In the present work, the use of a WAVE Bioreactor™ system 20/50 in perfusion mode with10 L disposable Cellbag™ bioreactors customized with two dip tubes in combination with disposable hollow fiber filters as external cell separating devices were investigated. A comparison between Alternating Tangential Flow (ATF) and Tangential Flow Filtration (TFF) was performed using a recombinant CHO cell line producing a monoclonal antibody (mAb) as a model system.

## Materials and methods

A research cell line DHFR^-^ CHO producing mAb was used. During expansion cultures in shake flasks, MTX selection pressure was performed but omitted in bioreactor. A WAVE Cellbag™ (10 L) containing two dip tubes (GE Healthcare) at 4L working volume was connected either to an ATF-2 device (Refine Technology, USA) or to a ReadyToProcess™ filter (TFF) via a Watson Marlow 620S pump. In both systems, the hollow fiber filters (HF), RTPCFP-2-E-4X2MS, GE Healthcare, Sweden, had 0.2 μm pore size, 1 mm lumen and 850 cm^2^ filter area. The pressure rising flow (ATF), the exhaust flow (ATF) and the recirculation flow rate (TFF) were 0.7 or 1 L/min. The set-points of DO, pH and temperature were 35 %, 7 and 37°C respectively. The agitation rate was varied between 20-26 rpm, 6-7° and 20-27 rpm, 6-8° for ATF and TFF respectively. O_2_ addition was performed in the headspace (20-100%) and the DO control was operated by varying the agitation.

The cells were cultivated in serum-free, animal-component free IS CHO CD XP medium (Irvine Scientific, USA) with hydrolysate, supplemented with 3 % of IS-CHO Feed-CD XP (Irvine Scientific, USA) and with 4 mM glutamine with a cell specific perfusion rate of 0.05 Reactor Volume/(day x 10^6^ cells/mL). Supplementations of glucose or glutamine were performed according to cell need. The pH was controlled by adding 0.5 M Na_2_CO_3_ or pulsing CO_2_ into the headspace. The cell density, viability, pH, pCO_2_, concentrations of glucose, lactate, glutamine and ammonia were measured by Bioprofile FLEX (Nova Biomedical). Antifoam C (Sigma Aldrich, US) was added up to 50 ppm concentration in the bioreactor either by boost addition or by continuous pumping. The mAb concentration was measured by protein A HPLC.

## Results

### Cell density, viability and perfusion rate

**Figure 1:**
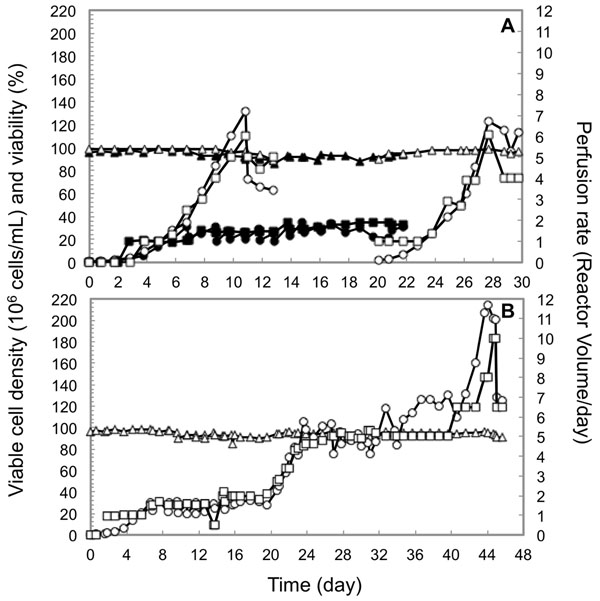
Viable cell density (●, ○), viability (▲, △) and perfusion rate (■, □) in perfusion processes using ATF system (A) and TFF system (B). Closed symbols indicate the ATF9 perfusion experiment, whereas the open symbols represent the ATF15 and TFF10 perfusion experiments.

Two perfusion experiments (ATF9 and ATF15) were conducted using ATF (Figure 1A) and one perfusion culture (TFF10) using TFF device (Figure 1B). In experiments ATF9 and TFF10, cell density was maintained between 20-30 x 10^6^ cells/mL by daily cell bleeds for 2 weeks using one HF. Interestingly, in experiment ATF9, the use of an ATF flow rate of 0.7 L/min was not sufficient to remove bubbles entrapped in the HF resulting in a decrease in viability (93.5% *vs.* 97%). Therefore, from day 9, the flow rate was increased to 1 L/min, *i.e.* shear stress of 3400 s^-1^. Experiment ATF15 was performed at 1 L/min.

In experiment ATF15, a cell density of 132 x 10^6^ cells/mL was reached after 10 days of culture of exponential growth. Reaching this density coincided with interruption of ATF function due to insufficient pressure to push the highly viscous cell broth, showing the limit of this system at this very high cell density when using a non-pressurisable disposable bioreactor. A batch culture was then carried out between days 13 and 20 (data not shown) after which perfusion was re-started. A maximal cell density of 123 x 10^6^ cells/mL was obtained confirming the first observed maximal cell density. The culture was then continued for 4 days at cell density around 100 x 10^6^ cells/mL by performing daily cell bleeds showing that healthy high cell density culture could be obtained somewhat under 130 x 10^6^ cells/mL in the same culture.

Using the TFF, exponential cell growth was obtained at similar rate as with ATF, the cell density was then maintained around 100 x 10^6^ cells/mL by daily cell bleeds for 2 weeks. Then, higher cell densities were achieved, up to more than 200 x 10^6^ cells/mL for 2 days. Reaching 214 x 10^6^ cells/mL coincided with the limit of this system due to high pressure in re-circulation loop (1 bar) related to the viscosity, oxygenation limitation and CO_2_ accumulation (31 kPa). During these runs, CHO cells were cultivated at high and very high cell densities with a cell viability maintained above 90% (mostly around 95%).

### Total mAb production

Over an 17-day period of culture with cell densities maintained at 20-30 x 10^6^ cells/mL, a total of 9 g and 7 g of mAb were harvested in the ATF9 and TFF10 processes respectively (Table 1). The lower yield obtained using the TFF was mainly due to mAb removed in cell bleeds (30% in TFF *vs.* 19% in ATF). In both cultures, the cell specific production rates of mAb were similar and around 10-15 pg/cell/day (data not shown).

**Table 1:** Comparison of mAb production using ATF or TFF system at day 17 with cell densities maintained at 20-30 x 10^6^ cells/mL.

	ATF	TFF
Production in harvest (g)	**9**	**7**
Yield (production in harvest/total production) (g/g in %)	**75**	**55**
Total removal of mAb in cell bleeds/total production (g/g in %)	**19**	**30**
Residual mAb mass in bioreactor/total production (g/g in %)	**6**	**15**

## Conclusions

A very high cell density of 100 x 10^6^ cells/mL was stably maintained in growing phase and at high viability by performing cell bleeds in a perfused WAVE Bioreactor™ using TFF or ATF cell separations. With the settings used here, the maximal cell density was limited to 214 x 10^6^ cells/mL using TTF and 132 x 10^6^ cells/mL using ATF. Using TFF, the cell density could not be further increased due to the limitations of membrane capacity (for the encountered high viscosity), oxygenation and CO_2_ level, and using ATF due to pressure limitation to push highly viscous fluid and use of non-pressurisable disposable bioreactor. Thus, the TFF system allowed reaching higher cell densities. To our knowledge, it is the first time that a CHO cell density of more than 200 x 10^6^ cells/mL was achieved in a wave-agitated bioreactor.

Higher retentions of mAb were observed using the TFF system than using the ATF system. In perfusion, the major effect of this retention was loss of mAb in the cell bleeds. Consequently, ATF system was more favourable for production at stable cell density maintained by cell bleeds.

The use of a disposable bioreactor equipped with a disposable separation device offers a solution alleviating technical and sterility challenges occurring in perfusion processes.

